# Involvement of Oligodendrocytes in Tau Seeding and Spreading in Tauopathies

**DOI:** 10.3389/fnagi.2019.00112

**Published:** 2019-05-28

**Authors:** Isidro Ferrer, Meritxell Aguiló García, Margarita Carmona, Pol Andrés-Benito, Benjamin Torrejón-Escribano, Paula Garcia-Esparcia, José Antonio del Rio

**Affiliations:** ^1^Department of Pathology and Experimental Therapeutics, University of Barcelona, Barcelona, Spain; ^2^Senior Consultant, Bellvitge University Hospital, IDIBELL (Bellvitge Biomedical Research Centre), Barcelona, Spain; ^3^CIBERNED (Network Centre of Biomedical Research of Neurodegenerative Diseases), Institute of Health Carlos III, Ministry of Economy and Competitiveness, Madrid, Spain; ^4^Institute of Neurosciences, University of Barcelona, Barcelona, Spain; ^5^Biology Unit, Scientific and Technical Services, Hospitalet de Llobregat, University of Barcelona, Barcelona, Spain; ^6^Molecular and Cellular Neurobiotechnology, Institute of Bioengineering of Catalonia (IBEC), Barcelona Institute for Science and Technology, Parc Científic de Barcelona, Barcelona, Spain; ^7^Department of Cell Biology, Physiology and Immunology, Faculty of Biology, University of Barcelona, Barcelona, Spain

**Keywords:** tau, tauopathies, seeding and spreading, AD, ARTAG, GGT, PiD

## Abstract

**Introduction:** Human tau seeding and spreading occur following intracerebral inoculation into different gray matter regions of brain homogenates obtained from tauopathies in transgenic mice expressing wild or mutant tau, and in wild-type (WT) mice. However, little is known about tau propagation following inoculation in the white matter.

**Objectives:** The present study is geared to learning about the patterns of tau seeding and cells involved following unilateral inoculation in the corpus callosum of homogenates from sporadic Alzheimer's disease (AD), primary age-related tauopathy (PART: neuronal 4Rtau and 3Rtau), pure aging-related tau astrogliopathy (ARTAG: astroglial 4Rtau with thorn-shaped astrocytes TSAs), globular glial tauopathy (GGT: 4Rtau with neuronal tau and specific tau inclusions in astrocytes and oligodendrocytes, GAIs and GOIs, respectively), progressive supranuclear palsy (PSP: 4Rtau with neuronal inclusions, tufted astrocytes and coiled bodies), Pick's disease (PiD: 3Rtau with characteristic Pick bodies in neurons and tau containing fibrillar astrocytes), and frontotemporal lobar degeneration linked to P301L mutation (FTLD-P301L: 4Rtau familial tauopathy).

**Methods:** Adult WT mice were inoculated unilaterally in the lateral corpus callosum with sarkosyl-insoluble fractions or with sarkosyl-soluble fractions from the mentioned tauopathies; mice were killed from 4 to 7 months after inoculation. Brains were fixed in paraformaldehyde, embedded in paraffin and processed for immunohistochemistry.

**Results:** Tau seeding occurred in the ipsilateral corpus callosum and was also detected in the contralateral corpus callosum. Phospho-tau deposits were found in oligodendrocytes similar to coiled bodies and in threads. Moreover, tau deposits co-localized with active (phosphorylated) tau kinases p38 and ERK 1/2, suggesting active tau phosphorylation of murine tau. TSAs, GAIs, GOIs, tufted astrocytes, and tau-containing fibrillar astrocytes were not seen in any case. Tau deposits were often associated with slight myelin disruption and the presence of small PLP1-immunoreactive globules and dots in the ipsilateral corpus callosum 6 months after inoculation of sarkosyl-insoluble fractions from every tauopathy.

**Conclusions:** Seeding and spreading of human tau in the corpus callosum of WT mice occurs in oligodendrocytes, thereby supporting the idea of a role of oligodendrogliopathy in tau seeding and spreading in the white matter in tauopathies. Slight differences in the predominance of threads or oligodendroglial deposits suggest disease differences in the capacity of tau seeding and spreading among tauopathies.

## Introduction

Tauopathies are progressive neurodegenerative diseases characterized by the accumulation of abnormal hyper-phosphorylated tau deposits in neurons and glial cells. These diseases are classified according to the clinical symptoms and neuropathological features, including particular regional and cellular vulnerability, together with biochemical and genetic determinants (Kovacs, 2015a,b). Biochemical factors are mainly defined by the accumulation of particular tau isoforms; protein tau in human brain (encoded by *MAPT* gene*)* is expressed in six isoforms arising from alternative splicing of exons 2 and 3 which encode N-terminal sequences, and exon 10 which encodes a microtubule-binding repeat domain; isoforms with 352 (3R/0N), 381 (3R/1N), and 410 (3R/2N) amino acids are 3Rtau; and isoforms with 383 (4R/0N), 412 (4R/1N), and 441 (4R/2N) amino acids are 4Rtau (Goedert et al., 1989, 1992; Spillantini and Goedert, 1998). Genetic factors largely depend on the mutation of *MAPT* which leads to familial frontotemporal lobar degeneration with tau deposits (fFTLD-tau).

The main sporadic tauopathies are Alzheimer's disease (AD), which is a 4Rtau+3Rtau plus β-amyloidopathy characterized by the combined accumulation of abnormal tau and β-amyloid in β-amyloid plaques and cerebral blood vessels in β-amyloid angiopathy (Duyckaerts and Dickson, 2011; Lowe and Kalaria, 2015); primary age related tauopathy (PART), a pure neuronal 3Rtau+4Rtau tauopathy; aging-related tau astrogliopathy (ARTAG), a pure 4Rtau astroglial tauopathy characterized by thorn-shaped astrocytes (TSAs) and granular/fuzzy astrocytes; globular glial tauopathy (GGT), a 4Rtau neuronal and glial tauopathy with distinctive globular astroglial and oligodendroglial inclusions (GAIs and GOIs, respectively); Pick's disease (PiD), a 3Rtau mainly neuronal tauopathy with some tau deposits in fibrillar astrocytes; progressive supranuclear palsy (PSP), a 4Rtau neuronal and glial tauopathy with characteristic tufted astrocytes (TAs) and coiled bodies; corticobasal degeneration (CBD), a 4Rtau neuronal and glial tauopathy with characteristic astrocytic plaques and coiled bodies; and argyrophilic grain disease (AGD), a 4Rtau neuronal and glial tauopathy with neuronal pre-tangles, grains in the neuropil, TSAs, and coiled bodies (Tolnay et al., 1997; Jellinger, 1998; Bigio et al., 2001; Ferrer et al., 2003, 2008, 2013; Powers et al., 2003; Piao et al., 2005; Josephs et al., 2006; Giaccone et al., 2008; Kovacs et al., 2008, 2016, 2017; Fu et al., 2010; Ahmed et al., 2011, 2013; Dickson et al., 2011; Muñoz et al., 2011; Tolnay and Braak, 2011; Crary et al., 2014; Duyckaerts et al., 2015; Jellinger et al., 2015; Ferrer, 2018a; Kovacs, 2018).

The main familial tauopathies are familial AD (fAD), linked to mutations in the genes encoding β-amyloid precursor protein (*APP)*; presenilin 1 (*PSEN1*) and presenilin 2 (*PSEN2*), with biochemical characteristics similar to those in sporadic AD (sAD) (Bertram and Tanzi, 2011); and familial frontotemporal lobar degeneration linked to tau mutations (fFTLD-tau), in which the clinical features, neuropathology, and biochemical attributes largely depend on the localization of the mutation in *MAPT* together with individual variations (Spillantini et al., 1997; Iseki et al., 2001; Muñoz and Ferrer, 2008; Spina et al., 2008; Ghetti et al., 2011; Tacik et al., 2016, 2017; Borrego-Écija et al., 2017).

One of the mechanisms involved in the progression of tauopathies is inter-cellular and trans-regional propagation of the altered protein tau (de Calignon et al., 2012; Liu et al., 2012; Iba et al., 2013; Dujardin et al., 2014; Peeraer et al., 2015; Stancu et al., 2015; Lewis and Dickson, 2016; Goedert and Spillantini, 2017; Mudher et al., 2017). Studies analyzing tau seeding and propagation *in vivo* have been performed following inoculation of human homogenates in mice expressing human tau (Clavaguera et al., 2009, 2013a,b, 2015; Ahmed et al., 2014; Boluda et al., 2015) or in WT mice (Audouard et al., 2016; Guo et al., 2016; Narasimhan et al., 2017). Homogenates are obtained from transgenic mice expressing human tau or, more commonly, from human neuronal or mixed neuronal and glial tauopathies; these studies are focused on neurons as main targets of tau propagation although glial cells are also involved (Clavaguera et al., 2009, 2013a,b, 2015; Ahmed et al., 2014; Boluda et al., 2015; Audouard et al., 2016; Guo et al., 2016; Narasimhan et al., 2017). However, tau seeding and spreading occurs in neurons, astrocytes, and oligodendrocytes following intrahippocampal inoculation of homogenates from pure ARTAG in WT mice (Ferrer et al., 2018), raising the important question of the relevance of astrocytes in the pathogenesis and progression of at least certain tauopathies. On the other hand, tau homogenates have been inoculated into different gray matter regions including cerebral cortex, hippocampus, striatum and locus ceruleus, among other gray matter centers. Little attention has been paid regarding direct and selective tau inoculation into the white matter.

In this line of thinking, and considering that (1) the white matter is involved, often severely, in the majority of tauopathies; and (2) the majority of cells in the white matter are oligodendrocytes, the present study has been designed to learn about the capacity of seeding and spreading of abnormal tau and the involvement of oligodendrocytes in the process, following unilateral inoculation of sarkosyl-insoluble and sarkosyl-soluble fractions from distinct human tauopathies, including AD, PART, ARTAG, GGT, PiD, PSP, and fFTLD-tau linked to P301L mutation (fFTLD-P301L or P301L), into the lateral corpus callosum of WT mice.

## Materials and Methods

### Human Cases

Brain tissue was obtained from the Institute of Neuropathology HUB-ICO-IDIBELL Biobank following the guidelines of Spanish legislation on this matter (Real Decreto de Biobancos 1716/2011) and approval of the local ethics committee. One hemisphere was immediately cut in coronal sections, 1 cm thick, and selected areas of the encephalon were rapidly dissected, frozen on metal plates over dry ice, placed in individual air-tight plastic bags, and stored at −80°C until use for biochemical studies. The other hemisphere was fixed by immersion in 4% buffered formalin for 3 weeks for morphological studies and neuropathological diagnoses.

Cases were categorized and selected following well-established neuropathological criteria (Braak et al., 2006; Dickson et al., 2011; Muñoz et al., 2011; Ahmed et al., 2013; Crary et al., 2014; Kovacs et al., 2016; Borrego-Écija et al., 2017) excluding cases with combined pathologies (excepting discrete small blood vessel disease related to age, and atherosclerosis), systemic diseases, and prolonged terminal hypoxia. It is worth stressing that ARTAG cases were pure forms without additional tau pathology excepting PART stage I-II (without involvement of the hippocampus). The cause of death was variable and included bronchopneumonia, rupture of aortic aneurysm, respiratory failure, cardiac arrest, kidney failure, pulmonary thromboembolism, and metastatic carcinoma. Post-mortem delay from death to tissue processing was between 4 h and 18 h. The final selection of inocula was based on the optimal profile of the western blot bands of sarkosyl-insoluble fractions visualized with anti-P-tauSer422 antibodies (see below). Selected samples were from sporadic AD (Braak stage VI/C: one man and one woman aged 82 and 76 years, respectively); PART (Braak stage IV: one man and one woman aged 68 and 72, respectively); pure ARTAG (two women aged 68 and 72, and one man 68 years old); GGT (one man and one woman aged 49 and 43 years, respectively); PiD (a 67-year-old man); PSP (one woman aged 72 years old); fFTLD-P301L (one man 53 years old); and one control (one man 61 years old).

### Sarkosyl-Insoluble and Sarkosyl-Soluble Fractions Used for Inoculations

Human brain samples used for brain inoculation in mice were obtained from the hippocampus in cases of AD, PART, PiD, fFTLD-P301L, and control; temporal white matter in cases of ARTAG; striatum in PSP; and prefrontal cortex area 8 in GGT cases.

Frozen samples of about 1 g were lysed in 10 volumes (w/v) with cold suspension buffer (10 mM Tris-HCl, pH 7.4, 0.8 M NaCl, 1 mM EGTA) supplemented with 10% sucrose, protease, and phosphatase inhibitors (Roche, GE). The homogenates were first centrifuged at 20,000×g for 20 min (Ultracentrifuge Beckman with 70Ti rotor), and the supernatant (S1) was saved. The pellet was re-homogenized in 5 volumes of homogenization buffer and re-centrifuged at 20,000×g for 20 min (Ultracentrifuge Beckman with 70Ti rotor). The two supernatants (S1 + S2) were then mixed and incubated with 0.1% N-lauroylsarkosynate (sarkosyl) for 1 h at room temperature while being shaken. Samples were then centrifuged at 100,000×g for 1 h (Ultracentrifuge Beckman with 70Ti rotor). Sarkosyl-insoluble pellets (P3) were re-suspended (0.2 ml/g) in 50 mM Tris–HCl (pH 7.4). Protein concentrations were quantified with the bicinchoninic acid assay (BCA) assay (Pierce, Waltham, MA). Sarkosyl-insoluble and sarkosyl-soluble fractions were frozen at −80°C until use.

### Western Blotting of Sarkosyl-Insoluble Fractions

Samples were mixed with loading sample buffer and heated at 95°C for 5 min. Sixty microgram of protein was separated by electrophoresis in SDS-PAGE gels and transferred to nitrocellulose membranes (200 mA per membrane, 90 min). The membranes were blocked for 1 h at room temperature with 5% non-fat milk in TBS containing 0.2% tween and were then incubated with the primary antibody, anti-tau Ser422 (diluted 1:1,000; Thermo Fisher (Waltham, MA, USA). After washing with TBS-T, blots were incubated with the appropriate secondary antibody (anti-rabbit IgG conjugated with horseradish peroxidase diluted at 1:2,000, DAKO, DE) for 45 min at room temperature. Immune complexes were revealed by incubating the membranes with chemiluminescence reagent (Amersham, GE Healthcare, Buckinghamshire, UK) (Ferrer et al., 2018).

### Animals and Tissue Processing

Wild-type C57BL/6 mice from our colony were used. All animal procedures were carried out following the guidelines of the European Communities Council Directive 2010/63/EU and with the approval of the local ethical committee (University of Barcelona, Spain). The age and number of animals, and the survival times, are listed in Table 1.

**Table 1:** Mice used in the study of unilateral inoculation in the lateral corpus callosum, age of inoculation, age of killing, survival, and type of inoculum; AD, Alzheimer's disease stage VI; PART, primary age-related tauopathy; ARTAG, aging related tau astrogliopathy; GGT, globular glial tauopathy; PSP, progressive supranuclear palsy; PiD, Pick's disease; and fFTLD-P301L, familial frontotemporal lobar degeneration linked to P301L mutation in *MAPT*.

**Age inoculationmonth**	**Age killed month**	**Survival months**	**Inoculum**	**Tau-positive oligodendrocytes in ipsilateral/contralateral corpus callosum**	**Tau-positive threads/dots in ipsilateral/contralateral corpus callosum**	**PLP1-immunoreactive globules and balls in ipsilateral/contralateral corpus callosum**
7	11	4	Control	–/–	–/–	–/–
			Control	–/–	–/–	–/–
			AD	++/–	ϕϕ/–	+/–
			ADs	–/–	–/–	–/–
			GGT	±	ϕ/–	–/–
			GGT	±	ϕ/–	–/–
			GGT	±	ϕ/–	–/–
			GGT	±	ϕ/–	–/–
			GGTs	–/–	–/–	–/–
			GGTs	–/–	–/–	–/–
			GGTs	–/–	–/–	–/–
12	18–19	6–7	AD	+++/+++	ϕϕ/ϕϕ	±
			AD	+++/+++	ϕϕ/ϕϕ	±
			PART	+++/+++	ϕϕ/ϕϕ	±
			PART	+++/+++	ϕϕ/ϕϕ	±
			PART	+++/++	ϕϕ/ϕ	±
			PART	+++/++	ϕ/ϕ	±
			ARTAG	+++/+	ϕϕ/ϕ	±
			ARTAG	+++/+++	ϕ/ϕ	±
			ARTAG	+++/++	ϕϕ/ϕ	±
			GGT	++/+	ϕϕ/ϕ	±
			GGT	++/++	ϕϕ/ϕϕ	±
			GGT	+/+	ϕϕ/ϕ	–/–
			GGT	–/–	–/–	–/–
			control	–/–	–/–	–/–
			control	–/–	–/–	–/–
			ARTAGs	–/–	–/–	–/–
			ARTAGs	–/–	–/–	–/–
10-12	16-18	6	PSP	++/+	ϕ/ϕ	±
			PSP	++/+	ϕ/ϕ	–/–
			PSP	+++/++	ϕ/ϕ	±
			PiD	+/+	ϕϕ/ϕ	–/–
			PiD	+/+	ϕϕ/ϕ	–/–
			PiD	+/+	ϕϕ/ϕ	±
			PiD	–/–	–/–	–/–
			fFTLD-P301L	+/+	ϕϕ/ϕ	±
			fFTLD-P301L	±	ϕϕ/ϕ	–/–
			fFTLD-P301L	+/+	ϕϕ/ϕ	–/–

*The column on the right is a summary of the efficiency of the injection referred to the presence of deposits in oligodendrocytes (percentage of tau-containing oligodendrocytes of the total number of oligodendrocytes per section) in the ipsilateral and contralateral corpus callosum in each mouse. Inoculum refers to sarkosyl-insoluble fractions of the corresponding human tauopathies with the exception of ADs, GGTs, and ARTAGs, which indicate sarkosyl-soluble fractions of AD, GGT, and ARTAG, respectively. Signs of the semiquantitative study: –, indicate no tau-positive oligodendrocytes; +, 5–9% tau-positive oligodendrocytes; ++, 10–39%, and +++, 40–65% tau-positive oligodendrocytes of the total number of oligodendrocytres per section as revealed with double-labeling immunofluorescence with Olig2 and AT8 antibodies and visualization with confocal microscopy. Tau-containing threads and dots along the corpus callosum were evaluated as: –, no deposits; ϕ, a few per section; ϕϕ, some per section; and ϕϕϕ, many per section. Regarding myelin alterations in PLP1-immunostained sections: –, no alterations; +, present*.

### Inoculation Into the Lateral Corpus Callosum

Mice were inoculated unilaterally with sarkosyl-insoluble fractions or with sarkosyl-soluble fractions from the above-mentioned tauopathies. In parallel, other mice were injected with 50 mM Tris-HCl (pH 7.4) as vehicle (negative) controls. Mice were deeply anesthetized by intra-peritoneal ketamin/xylazine/buprenorphine cocktail injection and placed in a stereotaxic frame after assuring lack of reflexes. Injections were done using a Hamilton syringe; the coordinates for lateral corpus callosum inoculations were: −1.9 AP; ± 1.4 ML relative to Bregma and −1.0 DV from the dural surface. A volume of 1.2 μl was injected at a rate of 0.1 μl/min. The syringe was retired slowly over a period of 10 min to avoid leakage of the inoculum. Each mouse was injected with inoculum from a single human case. Following surgery, mice were kept in a warm blanket and monitored until they recovered from the anesthesia. Carprofen analgesia was administered immediately after surgery and once a day during the following 2 days. Animals were housed individually with full access to food and water.

### Inoculation Into the Hippocampus of AD Homogenates

For comparative purposes two mice aged 10 months were inoculated with sarkosyl-insoluble fractions and two mice of similar age with sarkosyl-soluble fractions of AD cases into the hippocampus and killed at the age of 16 months following the same protocol. The coordinates for hippocampal injections were −1.9 AP; ± 1.4 ML relative to Bregma and −1.5 DV from the dural surface. A volume of 1.5 μl was injected at a rate of 0.05 μl/min in the hippocampus.

### Tissue Processing

Animals were killed under anesthesia and the brains were rapidly fixed with paraformaldehyde in phosphate buffer, and then embedded in paraffin. Consecutive serial sections, 4 μm thick, were obtained with a sliding microtome. De-waxed sections were stained with haematoxylin and eosin or processed for immunohistochemistry using the antibodies AT8 (directed against P-tau at Ser202/Thr205), PLP1 (directed against proteolipid protein 1) and RT97 (directed against neurofilaments of 200 kDa) Following incubation with the primary antibody, the sections were incubated with EnVision + system peroxidase for 30 min at room temperature. The peroxidase reaction was visualized with diaminobenzidine and H_2_O_2_. Control of the immunostaining included omission of the primary antibody; no signal was obtained following incubation with only the secondary antibody.

Double-labeling immunofluorescence was carried out on the de-waxed sections, 4μm thick, which were stained with a saturated solution of Sudan black B (Merck, DE) for 15 min to block autofluorescence of lipofuscin granules present in cell bodies, and then rinsed in 70% ethanol and washed in distilled water. The sections were boiled in citrate buffer to enhance antigenicity and blocked for 30 min at room temperature with 10% fetal bovine serum diluted in PBS. Then the sections were incubated at 4°C overnight with combinations of AT8 and one of the following primary antibodies: glial fibrillary acidic protein (GFAP), Iba-1, Olig2, and phospho-p38: p38-P (Thr180-Tyr182). Other sections were immunostained with anti-phospho-tauThr181 and anti-phospho ERK 1/2 (Thr202/Tyr204) (see Table 2 for the characteristics of the antibodies). After washing, the sections were incubated with Alexa488 or Alexa546 fluorescence secondary antibodies against the corresponding host species. Nuclei were stained with DRAQ5^TM^.

**Table 2:** : Characteristics of the antibodies used.

**Antibody**	**Mono-/polyclonal**	**Dilution**	**Supplier**	**Country**
Iba1	Rabbit polyclonal	1:1000	Wako	Richmond, VA, USA
phospho-tau Thr181	Rabbit polyclonal	1:50	Cell Signaling	Danvers, MA,USA
phospho-tau Ser422	Rabbit polyclonal	1:1000	Thermo Fisher	Waltham, MA, USA
AT8 (Ser202/Thr205)	Monoclonal	1:50	Innogenetics	Ghent, BE
glial fibrillary acidic protein (GFAP)	Rabbit polyclonal	1:500	Dako	Glostrup, DK
P38-P (Thr180-Tyr182)	Rabbit polyclonal	1:100	Cell Signaling	Danvers, MA, USA
ERK 1/2-P (Thr202/Tyr204)	Monoclonal	1:50	Merck-Millipore	Billerica, MA, USA
Olig2	Rabbit polyclonal	1:500	Abcam	Cambridge, UK
PLP1	Monoclonal	1:100	Lifespan Biosci	Seattle, WA, USA
RT97	Monoclonal	1:50	Novocastra, Leica Biosyst	Barcelona, Spain

The characteristics of the antibodies are listed in Table 2. Then the sections were mounted in Immuno-Fluore mounting medium, sealed, and dried overnight. Sections were examined with a Leica TCS-SL confocal microscope.

Semi-quantitative studies were carried in the ipsilateral corpus callosum and contralateral corpus callosum in three non-consecutive sections per case. Data were expressed as the percentage of oligodendrocytes (as revealed with the Olig2 antibody) with tau deposits (as seen with the antibody AT8) compared with the total number of oligodendrocytes in the same field following double-labeling immunofluorescence and examination with the confocal microcopy. Signs: –, indicates no tau deposits in oligodendrocytes; +, 5–9% tau-positive oligodendrocytes; ++, 10–39%, and + + +, 40–65% oligodendrocytes containing tau deposits from the total number of oligodendrocytes in the same field. Regarding the number of oligodendrocytes co-expressing phospho-tau and phospho-p38, data were expressed as the percentage of tau-positive oligodendrocytes containing active p38 from the total number of tau-containing oligodendrocytes in three non-consecutive sections in every case. Tau-containing threads and dots along the corpus callosum were evaluated as: –, no deposits; ϕ, a few per section; ϕϕ, some per section; and ϕ*ϕϕ*, many per section. Regarding myelin alterations in PLP1-immunostained sections: –, no alterations; +, present.

## Results

### Biochemical Characterization of Tau Inocula

**Figure 1:**
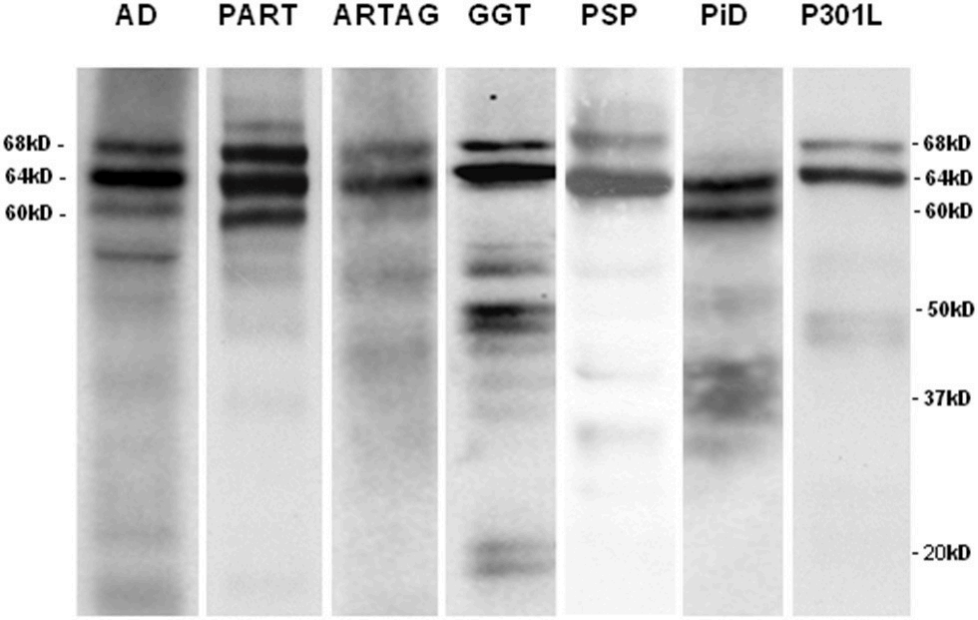
Sarkosyl-insoluble fractions of brain homogenates blotted with anti-tauSer422 in AD, PART, ARTAG, GGT, PSP, PiD, and fFTLD-P301L cases. The band pattern of AD and PART is characterized by three bands of 68, 64, and 60 kDa. AD shows, in addition, bands of low molecular weight some of them of about 20kDa and lower. The ARTAG case is characterized by two bands of 68 and 64 kDa. GGT shows bands of 68 and 64 kDa, bands between 50 and 37 kDa, and bands of truncated tau of about 20 kDa. PSP is characterized by two bands of 68 and 64 kDa whereas PiD is characterized by two bands of 64 and 60 kDa. Finally, fFTLD-P301L (P301L) shows two bands of 68 and 64 kDa.

Western blots of sarkosyl-insoluble fractions incubated with anti P-tau Ser422 antibodies showed the expected phospho-tau band pattern for each tauopathy. AD and PART were characterized by bands of 68, 64, and 60 kDa indicative of 3Rtau + 4Rtau tauopathies; longer exposure showed bands of 50 kDa and about 30–37 kDa, and lower bands of about 20 kDa. ARTAG, GGT, PSP, and fFTLD-P301L revealed two bands of 68 and 64 kDa specific to 4Rtau tauopathies. GGT also showed several bands of about 50 and 55 kDa, and lower bands of truncated tau of about 20 kDa. In contrast, PiD showed two bands of 64 and 60 kDa, distinctive of 3Rtau tauopathies, in addition to some smears of ~35 kDa (Figure 1).

### Tau Seeding and Spreading in Mice Inoculated in the Hippocampus and Lateral Corpus Callosum With Sarkosyl-Insoluble Fractions From AD

This set of experiments was used to show different regional vulnerability of the same type of homogenates when injected in the hippocampus with large numbers of neurons, and the corpus callosum with a major predominance of oligodendrocytes.

**Figure 2:**
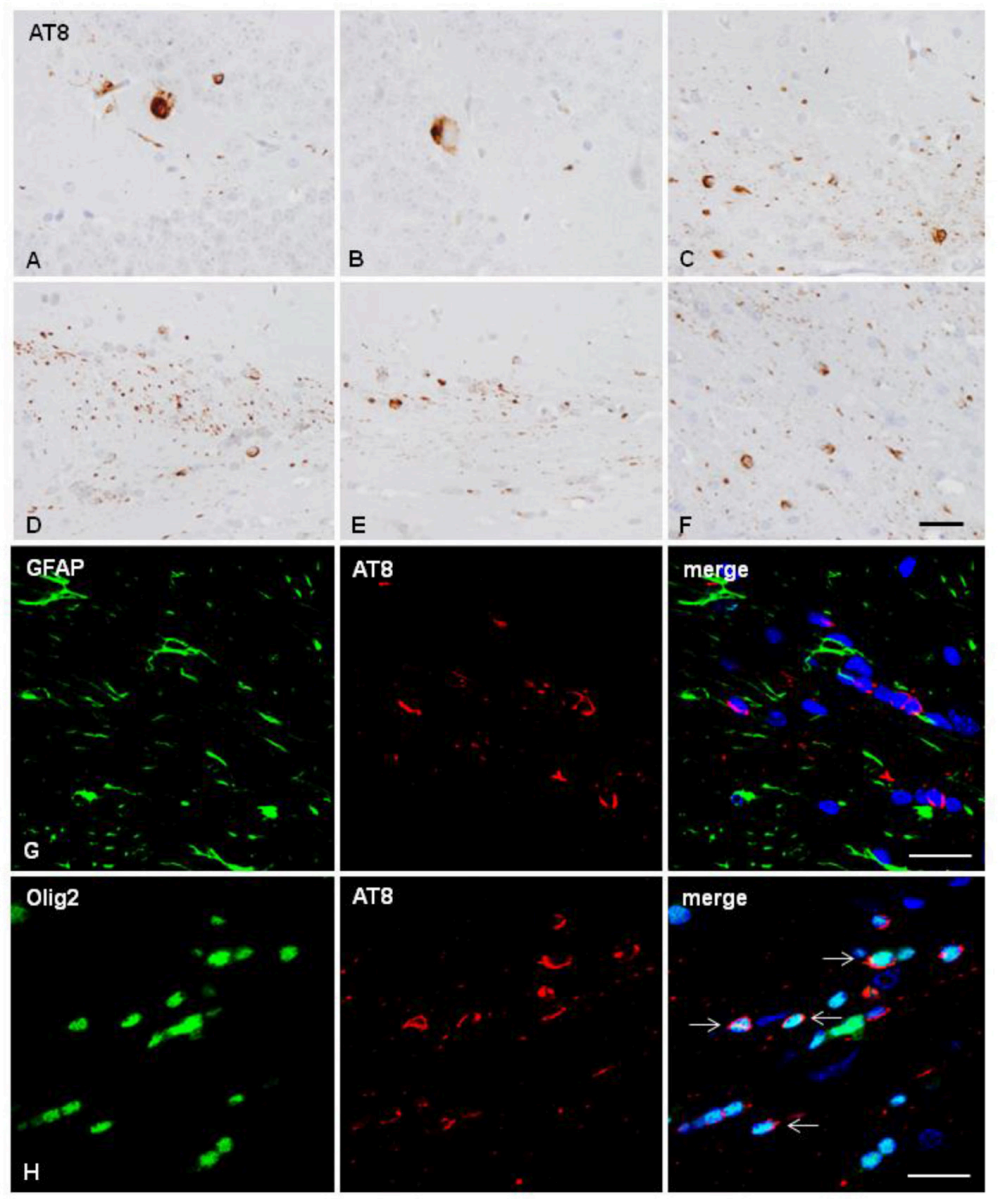
Hyper-phosphorylated tau containing cells and fibers following unilateral intra-hippocampal injection **(A–C)** and unilateral inoculation into the corpus callosum **(D–H)** of sarkosyl-insoluble fractions from AD cases into WT mice at the age of 10 and 12 months, respectively, and killed 6 months later. **(A,B)** tau-containing neurons and rare glial cells in the hippocampus; **(C)** tau-containing glial cells and threads in the fimbria. Tau- containing glial cells and threads in the ipsilateral **(D)**, middle region **(E)** and contralateral corpus callosum **(F)**. Paraffin sections immunostained with AT8 antibody and slightly counterstained with hematoxylin; **(A–F)**, bar = 25 μm. **(G,H)** Double-labeling immunofluorescence to GFAP (green) and AT8 (red) **(G)**, and to Olig2 (green) and AT8 (red) in the corpus callosum of WT mice inoculated with sarkosyl-insoluble fractions from AD at the age of 12 months and killed at the age of 18 months after unilateral inoculation in the corpus callosum. No tau deposits are seen in astrocytes **(G)**, but phospho-tau is present in oligodendrocytes (arrows). Paraffin sections, nuclei stained with DRAQ5™ (blue); bar = 20 μm.

Two mice unilaterally injected in the hippocampus with sarkosyl-insoluble fractions of AD at the age of 10 months and killed at the age of 16 months showed tau deposition in neurons and rare glial cells of the hippocampus (dentate gyrus and CA1 region), in glial cells in the fimbria (Figures 2A–C), and in some fibers and neurons in septal nuclei and periventricular hypothalamus (data not shown). One mouse unilaterally injected in the corpus callosum with sarkosyl-insoluble fractions from AD at the age of 7 months and killed at the age of 11 months showed phospho-tau deposits in the ipsilateral corpus callosum in threads and glial cells, and rarely extending to the middle corpus callosum (data not shown). Two mice unilaterally injected into the corpus callosum with sarkosyl-insoluble fractions of AD at the age of 12 months and killed at the age of 18 months showed tau deposition only in glial cells and threads of the ipsilateral (D), middle region (E) and contralateral (F) corpus (Figures 2D–F). Double-labeling immunofluorescence in the corpus callosum showed almost absent co-localization of GFAP and AT8 (Figure 2G), but numerous oligodendroglial cells with phospho-tau deposits (Figure 2H) (Table 1). No tau-positive deposits co-localized with the microglial marker Iba1 (not shown).

One mouse aged 7 months and two mice aged 10 months were inoculated with sarkosyl-soluble fractions from AD (ADs). No tau deposits were seen 4 months and 6 months, respectively, after the inoculation (Table 1).

### Tau Seeding and Spreading in Mice Inoculated in the Lateral Corpus Callosum With Sarkosyl-Insoluble and Sarkosyl-Soluble Fractions From Pure Tauopathies

Inoculation of sarkosyl-insoluble fractions obtained from pure tauopathies PART, ARTAG, GGT, PSP, PiD, and fFTLD-P301L was very effective as seen in Table 1. Of the 25 inoculated animals in the corpus callosum, only two (one inoculated with GGT and another with PiD) did not show phospho-tau deposits. Control cases (two mice inoculated at the age of 7 months and killed at the age 11 months, and two mice inoculated at the age 12 months and killed at the age of 18–19 months) were negative, as expected (Table 1).

Mice inoculated with sarkosyl-soluble fractions from GGT and ARTAG cases (GGTs and ARTAGs) did not show tau deposits at 4 months and 6 months, respectively, after unilateral inoculation into the corpus callosum (Table 1).

**Figure 3:**
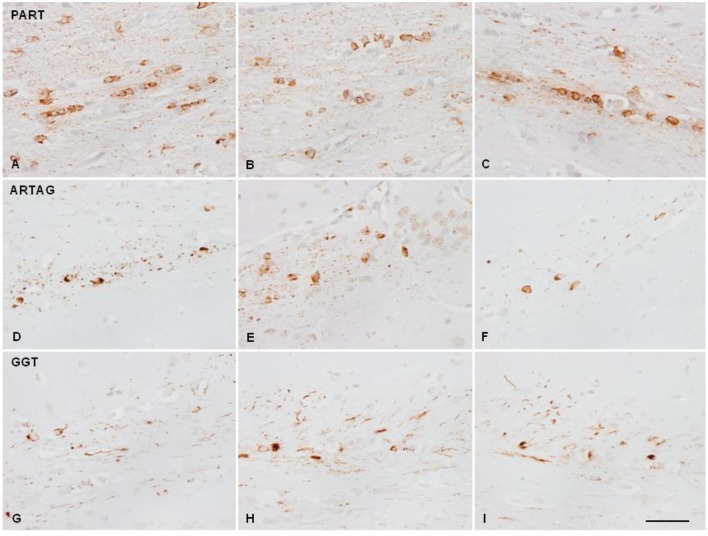
Hyper-phosphorylated tau-containing cells and threads following unilateral inoculation of sarkosyl-insoluble fractions into the lateral corpus callosum from PART, ARTAG, and GGT cases in WT mice at the age of 12 months and killed 6–7 months later; **(A,D,G)** correspond to the injected corpus callosum; **(B,E,H)** to the middle region of the corpus callosum; and **(C,F,I)** to the contralateral corpus callosum. Paraffin sections immunostained with antibodies AT8 slightly counterstained with hematoxylin; bar = 25 μm.

**Figure 4:**
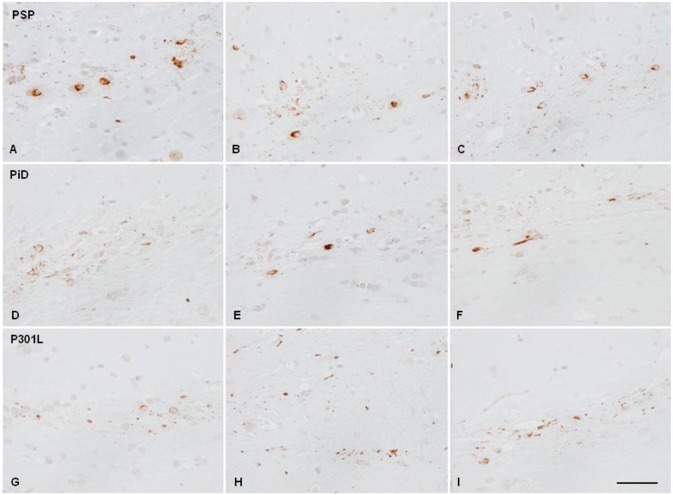
Hyper-phosphorylated tau-containing cells and threads following unilateral inoculation of sarkosyl-insoluble fractions into the lateral corpus callosum from PSP, PiD, and fFTLD-P3101L in WT mice at the age of 10–12 months and killed at the age of 16–18 months; **(A,D,G)** correspond to the injected corpus callosum; **(B,E,H)** to the middle region of the corpus callosum; and **(C,F,I)** to the contralateral corpus callosum. Paraffin sections immunostained with antibodies AT8 slightly counterstained with hematoxylin; bar = 25 μm.

Mice injected with sarkosyl-insoluble fractions from GGT at the age of 7 months and killed 4 months later showed phospho-tau deposits in the ipsilateral corpus callosum in threads and glial cells, rarely extending to the middle corpus callosum. Mice inoculated at the age of 10–12 months and surviving 6 to 7 months showed phospho-tau deposition in threads and glial cells in the ipsilateral corpus callosum, middle region and throughout the contralateral corpus callosum. The pattern was similar using PART, ARTAG, GGT, PSP, PiD, and fFTLD-P301L sarkosyl-insoluble fractions of brain homogenates, although with disease differences regarding tau immunostaining. PART and ARTAG homogenates showed the most dramatic capacity for phospho-tau labeling of oligodendrocytes followed by PSP. Phospho-tau in oligodendrocytes was less marked in GGT, PiD and fFTLD-P301L when compared with the other tauopathies (Figures 3, 4; Table 1). The morphology of tau deposits in glial cells was perinuclear, with certain coma-like enlargements in some cells, mimicking coiled bodies in human tauopathies; labeled cells were commonly arranged as oligodendrocyte rows. Glial cells with the morphology of thorn-shaped astrocytes (TSAs), globular astrocytic inclusions (GAIs), tufted astrocytes (TAs), and fibrillary astrocytes were not visualized in any cases. Importantly, globular oligodendroglial inclusions (GOIs) were not observed following inoculation of sarkosyl-insoluble fractions from GGT homogenates.

In addition to glial cells, tau-immunoreactive threads and dots along nerve fibers were observed in every positive case. However, threads were more common in GGT, PiD, and fFTLD-P301L when compared with the other tauopathies (Table 1).

Tau deposits were restricted to the corpus callosum, no neurons and other cells were stained in the contralateral cortex or in any other region at 6–7 months after inoculation into the corpus callosum.

### Identification of Hyper-Phosphorylated Tau-Containing Cells Using Double-Labeling Immunofluorescence and Confocal Microscopy

Double-labeling immunofluorescence was carried out using monoclonal anti-phospho-tau (clone AT8) and rabbit polyclonal antibodies to microglia (Iba1), astrocytes (GFAP), and oligodendroglia (Olig2).

**Figure 5:**
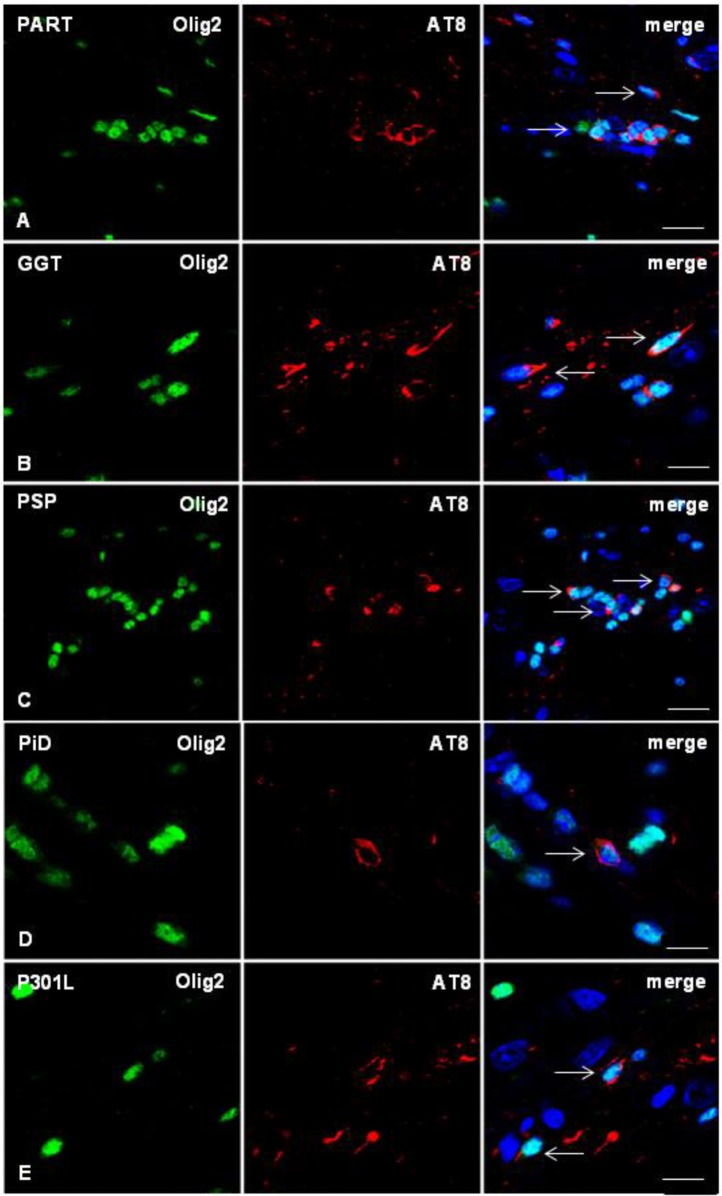
Double-labeling immunofluorescence to Olig2 (green) and AT8 (red) in the corpus callosum of WT mice inoculated with sarkosyl-insoluble fractions from PART, GGT, PSP, PiD, and FTLD-P301L and killed 6–7 months after unilateral inoculation showing phospho-tau deposition in oligodendrocytes (arrows). Paraffin sections, nuclei stained with DRAQ5™ (blue); **(A)** bar = 15 μm; **(B,D,E)**, bar = 10 μm; **(C)** bar = 20 μm.

**Figure 6:**
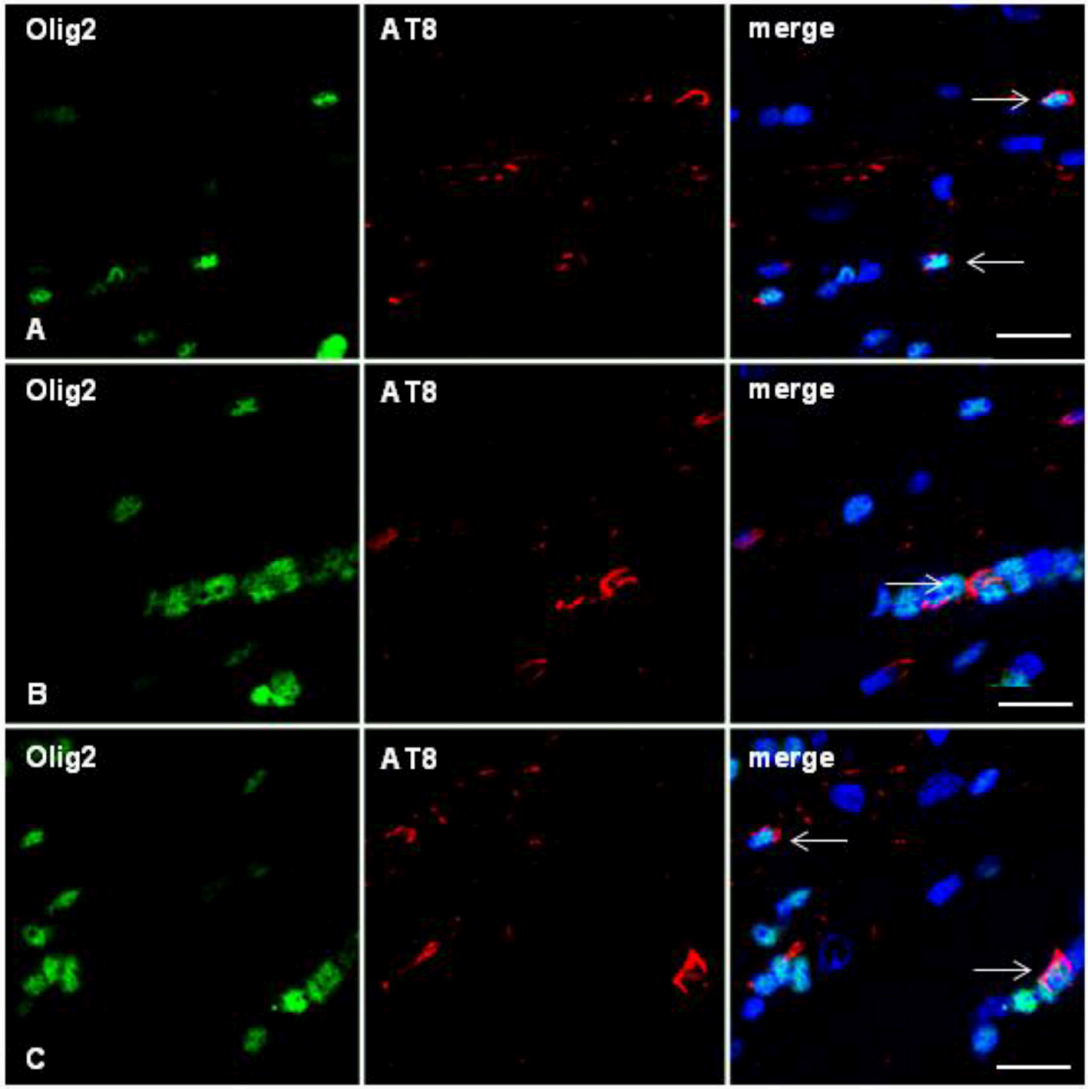
Double-labeling immunofluorescence to Olig2 (green) and AT8 (red) in the corpus callosum of WT mice inoculated with sarkosyl-insoluble fractions from ARTAG at 12 months and killed at 19 months showing oligodendrocytes (arrows) containing hyper-phosphorylated tau in the injected corpus callosum **(A)**, middle region **(B)**, and contralateral corpus callosum **(C)**. Paraffin sections, nuclei stained with DRAQ5™ (blue); bar = 15 μm.

As in the case of inoculation of AD sarkosyl-insoluble fractions, no tau deposits were seen in microglia at any age (mice with short survival: 4 months; and mice with long survival: 6–7 months). Phospho-tau deposits in astrocytes were rarely seen only in ARTAG, as detailed in a previous work (Ferrer et al., 2018). The vast majority of tau-containing cells in the corpus callosum were oligodendrocytes, independently of the tauopathy (Figure 5). The morphology of tau deposits was similar in all the tauopathies: PART, ARTAG, GGT, PSP, PiD, and fFTLD-P301L. The distribution of tau was perinuclear, forming caps or coma-like deposits, the latter resembling coiled bodies (Figure 5). Phospho-tau-labeled oligodendrocytes were found in the ipsilateral corpus callosum, middle region and contralateral corpus callosum (Figure 6). Semi-quantitative studies were carried out in double-immunolabeled sections using Olig2 and AT8 antibodies in three non-consecutive sections per case. Data were expressed as the percentage of oligodendroglia with tau deposits compared with the total number of oligodendrocytes in the same field. As summarized in Table 1, the percentage of labeled oligodendrocytes in the ipsilateral and contralateral hippocampus was higher in AD, PART and ARTAG than in GGT, and lower in PiD- and P301L-inoculated mice.

### Tau in Oligodendrocytes Is Associated With Activation of Tau-kinases in Tau-Positive Cells

**Figure 7:**
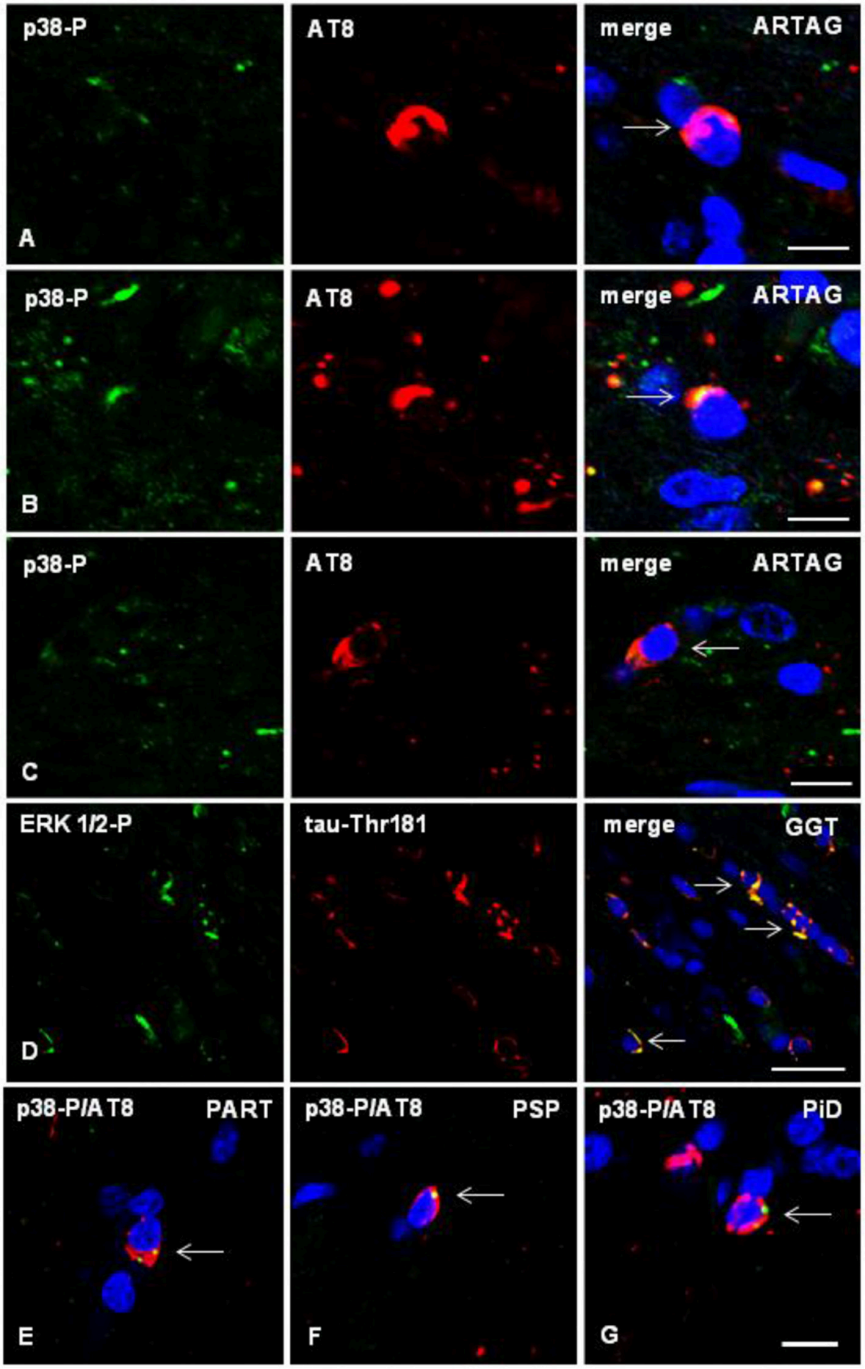
**(A–C)** Double-labeling immunofluorescence to phosphorylated p38 (p38-P: Thr180-182) (green) and AT8 (red) in the corpus callosum of WT mice inoculated unilaterally with sarkosyl-insoluble fractions from ARTAG at the age of 12 months and killed at the age of 19 months. Phospho-p38 kinase (p38-P) co-localizes with tau deposits (arrows) in oligodendrocytes in the ipsilateral corpus callosum **(A)**, middle region **(B)**, and contralateral corpus callosum **(C)**. **(D)** Double-labeling immunofluorescence to phospho-ERK 1/2 (Thr202/Tyr204) (green) and phospho-tau Thr181 (red) in the corpus callosum of WT mice inoculated with sarkosyl-insoluble fractions from GGT at the age of 12 months and killed at the age of 19 months. Phospho-ERK 1/2 co-localizes with tau deposits (arrows) in oligodendrocytes. **(E–G)** Double-labeling immunofluorescence to p38-P and AT8 (merge) in oligodendroglial cells of the corpus callosum of WT mice inoculated unilaterally with sarkosyl-insoluble fractions from PART **(E)**, PSP **(F)**, or PiD **(G)** at the age of 10–12 months and killed 6 months later. Paraffin sections, nuclei stained with DRAQ5™ (blue), **(A,B)**, bar = 5 μm; **(C)** bar = 10 μm; **(D)** bar = 20 μm; **(E–G)**, bar in **(G)** = 10 μm.

Double-labeling immunofluorescence with anti-phospho-tau antibodies (AT8) and antibodies directed to phosphorylated p38 kinase (p38-P Thr180-Tyr182), examined with confocal microscopy, identified co-localization of tau and active p38 (p38-P) in oligodendrocytes in mice inoculated with sarkosyl-insoluble fractions from tauopathies. Co-localization occurred in oligodendrocytes in the ipsilateral corpus callosum, middle region and contralateral corpus callosum (Figures 7A–C). Semiquantitative studies showed that between 20 and 30% of tau-containing oligodendrocytes co-localized phosphorylated p38 kinase.

Similarly, double-labeling immunofluorescence with anti-phospho-tauThr181 and phospho-ERK 1/2 (Thr202/Tyr204) showed co-localization of tau and phospho-ERK 1/2-P in oligodendrocytes in the corpus callosum of mice inoculated with sarkosyl-insoluble fractions from tauopathies (Figure 7D). The number of oligodendrocytes co-localizing phospho-tau and phospho-ERK 1/2 was between 25 and 35% of the total number of tau-containing oligodendrocytes.

The same pattern was seen in the different tauopathies (Figures 7E–G).

No expression of phospho-p38 and phospho-ERK 1/2 was observed outside the sites of phospho-tau deposits.

### Long-Term Effects on Myelin Linked to Tau Spreading

To learn whether tau deposition in oligodendrocytes and threads had any impact on myelin and nerve fibers, consecutive sections to those used for AT8 immunohistochemistry were immunostained with anti-PLP1 antibody, as a marker of myelin, and with the antibody RT97 as a marker of neurofilaments 200 kDa.

**Figure 8:**
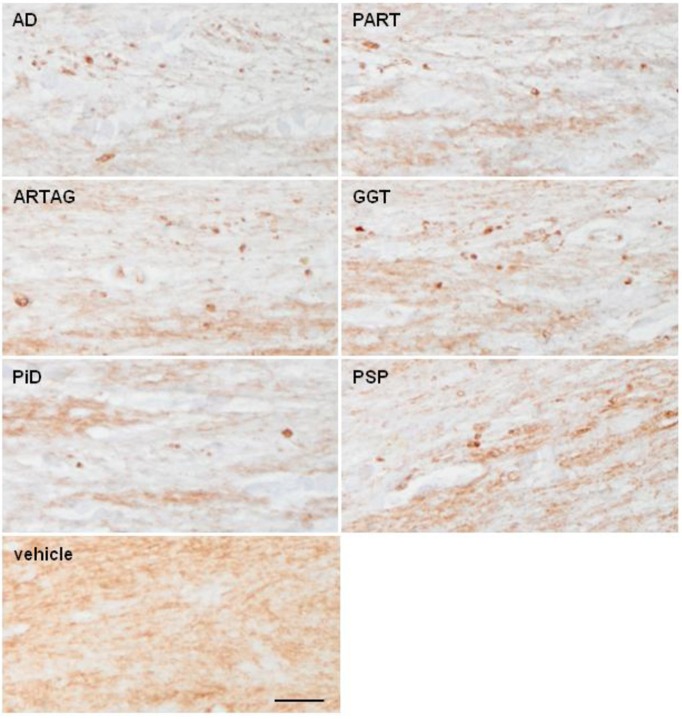
Representative sections of the corpus callosum immunostained with antibodies anti-PLP1 (proteolipid protein 1) in WT mice following inoculation of AD, PART, ARTAG, GGT, PiD, and PSP in the lateral corpus callosum at the age of 10–12 months and killed 6 months later. Disrupted myelin with occasional formation of small PLP1-immunoreactive balls is observed, when compared with mice inoculated with vehicle (control). Paraffin sections immunostained with antibodies PLP1 slightly counterstained with hematoxylin; bar = 50 μm.

Myelin lesions in the corpus callosum were very rare in inoculated animals killed 4 months after the injection. However, PLP1 immunohistochemistry disclosed slight myelin disruption and the presence of small globules and balls in the ipsilateral corpus callosum 6 months after the inoculation in some cases. These changes were observed in all samples in all tauopathies with tau deposits, although with disease-dependent variability; lesions were more common following inoculation of AD, PART, and ARTAG followed by GGT than following inoculation of homogenates from PSP, PiD, and fFTLD-P301L (Figure 8; Table 1). No changes were seen in cases inoculated with sarkosyl-soluble fractions and in controls.

In contrast to myelin alterations, no modifications were observed in parallel sections immunostained with the antibody RT97 (data not shown).

## Discussion

The present findings are in line with previous observations concerning tau seeding and spreading of abnormal tau derived from human brain homogenates of different tauopathies inoculated into the brain of mice (Clavaguera et al., 2009, 2013a,b, 2015; Ahmed et al., 2014; Boluda et al., 2015; Audouard et al., 2016; Guo et al., 2016; Narasimhan et al., 2017; Ferrer et al., 2018). In all these experimental paradigms, studies are focused on the neuronal involvement following inoculation in different regions of the gray matter including cerebral cortex, hippocampus, striatum and locus ceruleus, among others. However, the effects of inoculation of abnormal tau in the white matter have not been examined in detail.

The present observations show tau seeding and spreading in the corpus callosum of WT mice following inoculation of homogenates from AD (4R+3R tauopathy + β-amyloidopathy), and pure neuronal 4R+3R tauopathy (PART), pure astrocyte 4R tauopathy (ARTAG), combined neuronal and glial 4R tauopathy (PSP), neuronal and glial 4R tauopathy with specific globular glial inclusions (GGT), 3R tauopathy (PiD), and familial FTLD-P301L. In all these paradigms, oligodendrocytes, and threads are the main if not the only targets of abnormal tau, albeit with variations in the capacity for seeding and spreading, depending on the tauopathy.

The overwhelming presence of tau in oligodendrocytes in comparison to astrocytes following abnormal tau inoculation into the corpus callosum may be explained because oligodendrocytes represent about 75.4 ± 5.1% of cells in the human corpus callosum, (Yeung et al., 2014), and the great majority of oligodendrocytes in mouse corpus callosum are not replaced during the animal's lifetime (Tripathi et al., 2017). Other targets of tau seeding and spreading, as neurons in gray matter regions, are well documented (Clavaguera et al., 2009, 2013a,b, 2015; Ahmed et al., 2014; Boluda et al., 2015; Audouard et al., 2016; Guo et al., 2016; Narasimhan et al., 2017; Ferrer et al., 2018), and here supported as complementary data using the same homogenates of AD employed for callosal inoculation following injection into the hippocampus; inoculation of homogenates into the hippocampus produced tau deposition in neurons and their projections, in addition to glial cells in the fimbria.

Slight peculiarities in relation with the amount of labeled oligodendrocytes and threads in the analyzed tauopathies are probably related to particular characteristics of tau in the different diseases. This possibility is in agreement with the observation that distinct artificially-generated strains of tau produce different types of neuronal and glial inclusions depending on the strain (Sanders et al., 2014; Kaufman et al., 2016).

Morphological characteristics of tau inclusions in oligodendrocytes in the present experiments are reminiscent to coiled bodies which are the most typical tau oligodendroglial inclusions in the majority of human tauopathies. However, coiled-like bodies are also observed following inoculation of AD and PART sarkosyl-insoluble homogenates thus indicating that pure neuronal tauopathies (AD and PART) have the capacity to induce tau seeding and spreading in oligodendrocytes in WT mice. On the other hand, GOIs, which are typical of GGT, are not seen following inoculation of sarkosyl-insoluble homogenates from GGT cases. Based on these findings, it may be suggested that, in addition to the postulated tau strains, the characteristics of the host tau and the region of inoculation help determine the characteristics of tau seeding and spreading of human tau inoculated into WT mice. Therefore, the present observations show that inoculation of homogenates from specific tauopathies into the corpus callosum does not replicate important aspects of the corresponding human tauopathies. Regarding astrocytes, TSAs typical of ARTAG, GAIs typical of GGT, tau-containing fibrillary astrocytes found in PiD, and TA of PSP are not reproduced in WT following intracallosal inoculation of homogenates from the corresponding tauopathies. This may be due, in part, to the predominance of GAIs and TAs in gray matter regions in human diseases, while TSAs are typically localized in the white matter.

Importantly, sarkosyl-soluble fractions have no capacity of tau seeding in any cases.

It may be suggested that the progressive appearance of threads along the corpus callosum is due to diffusion of the seeds. However, it can be posed that tau seeding and spreading in oligodendrocytes is an active process. On the one hand, inoculated tau has a half-life of a few days (Guo et al., 2016), whereas tau immunostaining in the corpus callosum extends over greater distances with longer survival time. Moreover, abnormal tau deposits in inoculated mice particularly those in oligodendrocytes co-express phospho-kinase p-38 and phospho-ERK-1/2; expression of phospho-kinase p38 and phospho-ERK-1/2 is restricted to the regions with phospho-tau deposits including threads and dots, thus suggesting active phosphorylation of resident murine tau (Ferrer et al., 2001a,b, 2005; Ferrer, 2004; Puig et al., 2004).

Coiled bodies are present in most non-AD tauopathies, and the white matter is affected in the majority of tauopathies. Various transgenic mice expressing tau mutations have oligodendroglial, in addition to neuronal and astroglial inclusions (Götz et al., 2001; Lin et al., 2003, 2005; Ren et al., 2014; Ferrer, 2018b). Moreover, selective over-expression of mutant tau in oligodendrocytes using CNP promoter in mice produces filamentous inclusions in oligodendrocytes and progressive impairment of axonal transport, followed by myelin and axonal disruption (Higuchi et al., 2005). Finally, *in vitro* studies have also shown deleterious effects of abnormal tau expression and deposition in oligodendrocytes which are causative of degeneration in particular settings (Richter-Lansberg, 2008; Richter-Landsberg, 2016). Functional deficiencies linked to phospho-tau deposition in oligodendrocytes and threads is also supported in the present study by the demonstration of slightly disrupted myelin and the occasional presence of PLP1-immunoreactive balls and dots in the ipsilateral corpus callosum following inoculation of sarkosyl-enriched fractions from AD, PART, ARTAG and less commonly with GGT, PSP, PiD, and fFTLD-301L.

Together, the present findings show that the white matter may be involved in tau seeding and spreading in a variety of experimentally-induced tauopathies. This is in accordance with the well-recognized, although often minimized, tau involvement of the white matter in most human tauopathies. Moreover, the present observations point to oligodendrocytes as targets of tau seeding and spreading in the white matter, thus highlighting oligodendrogliopathy (Ferrer, 2018b) as a component in the pathogenesis of tauopathies.

## Data Availability

All datasets generated for this study are included in the manuscript and/or the supplementary files.

## Ethics Statement

Brain tissue was obtained from the Institute of Neuropathology HUB-ICO-IDIBELL Biobank following the guidelines of Spanish legislation on this matter (Real Decreto de Biobancos 1716/2011) and approval of the local ethics committee.

Wild-type C57BL/6 mice from our colony were used. All animal procedures were carried out following the guidelines of the European Communities Council Directive 2010/63/EU and with the approval of the local ethical committee (University of Barcelona, Spain).

## Author Contributions

MA and PG-E carried out the inoculations of the animals. PA-B and MC prepared the inocula, processed brain tissue and checked tissue lesions. BT-E obtained confocal microscopy images. IF and JdR designed the experiments and summarized the main conclusions. IF examined the brains of all inoculated animals, interpreted the lesions, and wrote the manuscript which was circulated for approval by all the authors.

### Conflict of Interest Statement

The authors declare that the research was conducted in the absence of any commercial or financial relationships that could be construed as a potential conflict of interest.
